# Cardioplegia at subnormothermia facilitates rapid functional resuscitation of hearts preserved in SOMAH for transplants

**DOI:** 10.1186/s13019-014-0155-z

**Published:** 2014-09-20

**Authors:** Samar K Lowalekar, Patrick R Treanor, Hemant S Thatte

**Affiliations:** Cardiothoracic Surgery Division, Brigham and Women's Hospital, VA Boston Healthcare System, 1400 V. F. W. Parkway, West Roxbury, Boston, 02132 Massachusetts USA; Department of Surgery, Brigham and Women's Hospital, VA Boston Healthcare System, Boston, Massachusetts USA; Harvard Medical School, Brigham and Women's Hospital, VA Boston Healthcare System, Boston, Massachusetts USA

**Keywords:** Cardioplegia, Transplantation, Reperfusion injury, High energy phosphates

## Abstract

**Objectives:**

Hearts preserved *ex vivo* at 4°C undergo time-dependent irreversible injury due to extreme hypothermia. Studies using novel organ preservative solution SOMAH, suggest that hearts are optimally `preserved' at subnormothermic temperature of 21°C. Present study evaluates relative efficacy of SOMAH `cardioplegia' at 4 and 21°C in preservation of optimum heart function after *in vitro* storage at subnormothermia.

**Methods:**

Porcine hearts arrested with SOMAH cardioplegia at 4 or 21°C were stored in SOMAH for 5-hour at 21°C (n = 5). At the end of storage, the weight of hearts was recorded and biopsies taken for cardiac tissue high energy phosphate level measurements. The hearts were then attached to a reperfusion apparatus and biochemical parameters including cardiac enzyme release and myocardial oxygen consumption and lactate production were determined in perfusate samples at regular intervals during ex vivo perfusion experiment. Functional evaluation of the hearts intraoperatively and ex vivo was performed by 2D echocardiography using trans-esophageal echocardiography probe.

**Results:**

Post-storage heart weights were unaltered in both groups, while available high-energy phosphates (HEP) were greater in the 21°C group. Upon ex vivo reperfusion, coronary flow was significantly greater (p < 0.05) in 21°C group. 2D echo revealed a greater cardiac output, fractional area change and ejection fraction in 21°C group that was not significantly different than the 4°C group. However, unlike 4°C hearts, 21°C hearts did not require inotropic intervention. Upon reperfusion, rate of cardiac enzyme release temporally resolved in 21°C group, but not in the 4°C group. 21°C working hearts maintained their energy state during the experimental duration but not the 4°C group; albeit, both groups demonstrated robust metabolism and function during this period.

**Conclusions:**

Rapid metabolic switch, increased synthesis of HEP, decreased injury and optimal function provides evidence that hearts arrested at 21°C remain viably and functionally superior to those arrested at 4°C when stored in SOMAH at ambient temperature pre-transplant.

**Ultramini-abstract:**

Cardioplegic arrest and preservation of hearts in SOMAH at ambient temperature efficiently conserves metabolism and function in *in vitro* porcine model of heart transplant.

**Electronic supplementary material:**

The online version of this article (doi:10.1186/s13019-014-0155-z) contains supplementary material, which is available to authorized users.

## Background

Cardioplegia, a technique to stop the heart intraoperatively, is an integral part of open-heart surgeries and is also a fundamental requisite in the field of heart transplant. During surgery it is important for cardioplegia solution to not only arrest the heart adequately but also prevent damage to cardiac tissue during and/or after infusion. Several strategies to attain this ideal situation have been attempted by altering cardioplegia temperature, or using blood, crystalloid or blood-crystalloid mixture that have produced equivocal results in their ability to arrest the heart [1]-[6]. However, the meta-analysis of these studies does not provide much evidence in favor of one or the other [7],[8]. Nevertheless, of all these strategies by far, the use of high K^+^ cardioplegia at extreme hypothermia (4°C) has become most widely accepted, especially in the procurement of donor hearts for transplant [9]. This is despite the growing awareness of the damaging effects of extreme hypothermia on solid organs [10],[11] and failure of presently used modalities in extracorporeal preservation of hearts to extend the *ex vivo* storage time beyond 4-6 hours.

Recent results from our laboratory using animal models of heart transplant have demonstrated that hearts preserved in SOMAH solution of our design, demonstrate greater accumulation of high energy phosphates (HEP) in comparison to the comparator solutions [12]-[16]. Furthermore, hearts preserved in SOMAH for 5-24 hours and not exposed to extreme hypothermia (4°C) demonstrate a robust maintenance of organ viability and function, and minimal injury upon reperfusion and reanimation [12]-[14]. Additionally, these studies also demonstrate that hearts stored in SOMAH at 21°C outperform those preserved at 4 and 13°C upon reanimation. However, in these investigations SOMAH cardioplegia was delivered at 4°C for arrest prior to excision of donor hearts. Since preservation of hearts in SOMAH at 21°C was superior than that at 4°C, the present study was undertaken to assess whether delivery of high K^+^ SOMAH cardioplegia at 21°C compared to that at 4°C would further augment and improve viability and energy metabolism, and also prevent injury in hearts stored *ex vivo* for 5 hours at 21°C, that were then reperfused and reanimated for evaluation of cardiac function.

## Methods

### Animal protocol

#### Heart procurement surgery and cardioplegia

Ten female Yorkshire swine (45-54 Kg) were used in this comparative study. Hearts were divided into two groups of either 4°C (n = 5) or 21°C cardioplegia (n = 5), as per the protocol approved by our Animal Studies Committee (Institutional Animal Care and Use Committee). Hearts were extracted using mediastinal approach as described [12]. The animals were bled from femoral vessels to collect blood for *ex vivo* experiments, and aorta was clamped when systolic pressure fell below 40 mmHg. 1000 ml of SOMAH cardioplegia (SOMAH [12] modified by addition of 20 mM K^+^, final concentration), at 4 or 21°C was infused into the aortic root at a pressure of 75-100 mmHg at a flow rate of 300-400 ml/minute using roller pump and pressure transducer (Myotherm Cardioplegia System, Medtronics, Minneapolis, MN, USA) and the data was recorded using iWorks system (Dover, NH, USA). After cardioplegic arrest, heart was dissected from all attachments and rinsed with normal saline before storing in SOMAH for 5-hours at 21°C. Hearts were transported to the lab within 15 minutes of excision.

#### Extracorporeal storage of heart

Hearts were placed in sterile zip-lock bags containing 2 L of SOMAH in water-jacketed water bath at 21 ± 2°C. The temperature of preservation solution was checked regularly during the entire storage period. Hearts were maintained in a non-contractile state by increasing SOMAH's plegia potential by supplementing the solution with 20 mM K^+^ complemented by 37 mM Mg^2+^[17],[18], during storage. Hearts in each group were weighed prior to and after 5-hour storage upon carefully emptying the heart chambers. Tissue punch biopsies (2X4 mm) were taken in the lab from the posterior wall of LV, 15 minutes into (0 hour; control) and after 5 hour storage for HEP assays.

### ATP and creatine phosphate assay

ATP and creatine phosphate (CP) were measured in tissue extracts as described [12],[19]. In brief, tissue biopsies were flash frozen and stored at -80°C; 20 mg of tissue was suspended in 400 μl of 0.4 M ice-cold perchloric acid and homogenized twice for 30 seconds. Homogenate was centrifuged at 1970 g for 10 minutes at 0°C. An aliquot of supernatant was neutralized with equal volume of ice-cold 0.4 M KHCO_3_ and centrifuged as above. The supernatant was stored at -80°C for ATP and CP measurements. The pellet was dissolved in equal volume of 0.1 M NaOH, centrifuged and used for protein assay. ATP and CP were measured using a bioluminescent assay kit (Sigma-Aldrich and GloMax-Multi + Detection System, Promega), according to the protocol provided by the manufacturer.

### Preparation of heart for ex vivo resuscitation and functional studies

Aorta and pulmonary artery (PA) were separated. Aorta was cannulated (1/2-3/8 inch tubing connector) and coronaries were gently flushed with 100 ml of SOMAH in both 4°C and 21°C cardioplegia groups at 40-50 mmHg pressure, carefully avoiding entry of air into the aorta. Pulmonary veins (PV) were separated and cannulated with 1/2-1/4 inch tubing connector. PA was cannulated for sample collection while superior and inferior vena cavas were ligated.

### Preparation of blood for ex vivo studies

Systemically heparinized blood was collected intraoperatively, leukodepleted (Pall Leukoguard filter) and stored at 4°C. Prior to experiments, *perfusate* was prepared by adjusting the hematocrit of blood to 20% using SOMAH solution (1:1 ratio to reduce viscous strain on heart) and warmed to 21°C. The perfusate, pH, glucose, K^+^, Ca^2+^ and HCO^3-^ were adjusted for swine blood levels (7.5; 100 mg/dl; 3.7, 1.38, and 32 mmol/l respectively), using 10% dextrose, KCl, CaCl_2_ and NaHCO_3_, respectively, as required.

### The SOMAH device

**Figure 1:**
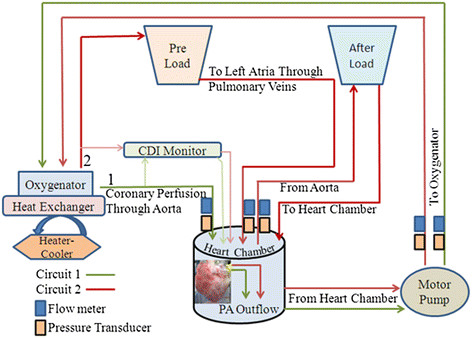
***Circuit diagram of the SOMAH Device.*** A custom-built apparatus was designed for extra-corporeal reanimation of hearts. The circuit 1 (green) visualizes antegrade coronary perfusion through aorta during initial reperfusion of hearts and circuit 2 (red), for perfusion of hearts through PV in the working state. In circuit 1, the perfusate is pumped from heart chamber to oxygenator-heat-exchanger system and eventually into aorta for coronary perfusion. The return of perfusate to heart chamber through PA completes the circuit. In circuit 2, blood pumped from heart to oxygenator-heat-exchanger system is collected in pre-load bag from where it drained into the PV by gravity. The pressures/flows are adjusted by altering the height of pre-load bag. This circuit is diverted into two components. First component is the part of perfusate that enters coronaries and returns to heart chamber through PA. The second component is formed by the perfusate that continues through aorta into the after-load chamber, from where the perfusate returns to heart chamber by gravity. A CDI monitor was incorporated beyond oxygenator-heat-exchanger system for real-time monitoring of perfusate pH, temperature, pO_2_, pCO_2_, K^+^ and HCO^3-^. Pressures and flows were recorded at various points in the two circuits and monitored using HMI software specifically written for SOMAH Device.

**Figure 2:**
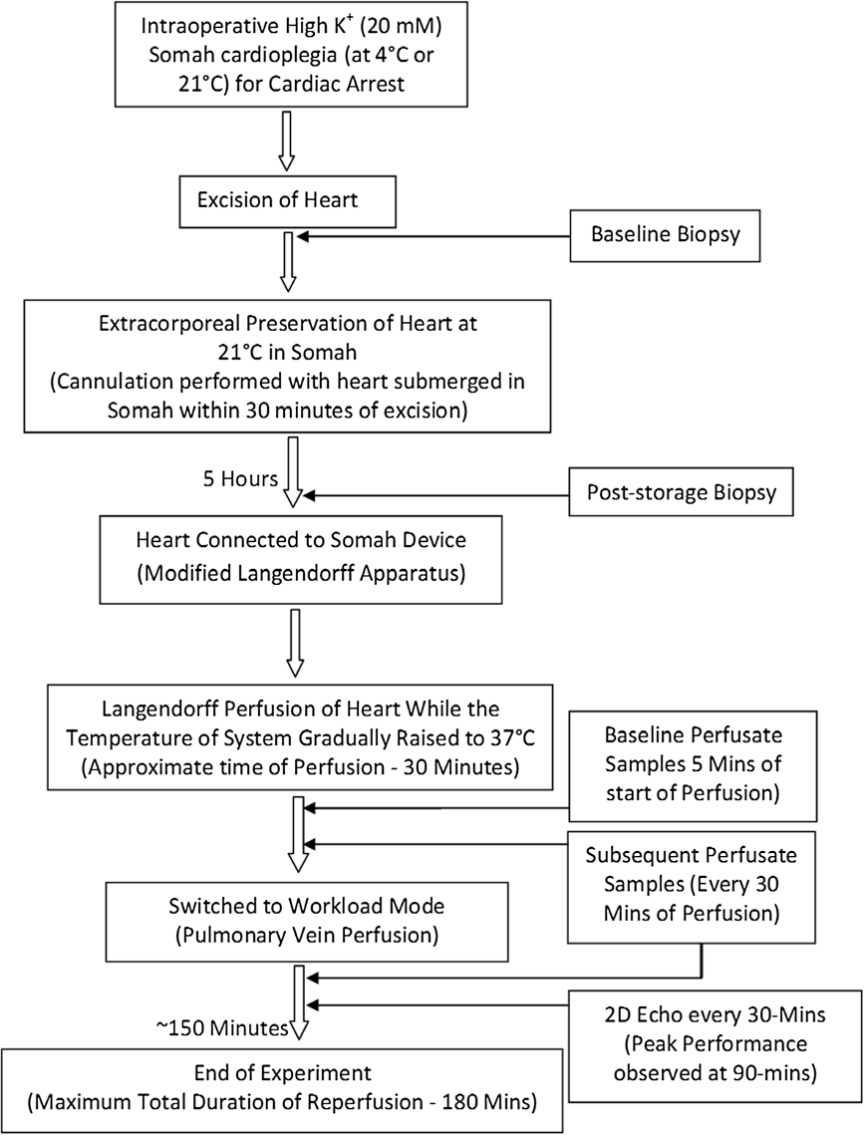
***Flow diagram of experimental design.*** Illustration shows the general experimental design of this study, starting from intraoperative cardioplegia for cardiac arrest to the end of ex vivo heart reperfusion experiment.

A custom-built apparatus was used for extra-corporeal reanimation of hearts (Figure 1, Additional file 1: Video). CDI monitor (Clinical Documentation Improvement monitoring system 500, Terumo cardiovascular systems corporation, Ann Arbor, MI), was used for real-time monitoring of perfusate pH, temperature, pO_2_, pCO_2_, K^+^ and HCO_3_^-^. These parameters were also analyzed in inflow/outflow samples using i-STAT analyzer (Abaxis Ltd, Union city, CA). Pressures and flows were recorded at various points in the circuits (Figure 2). A trans-esophageal echocardiography (TEE) probe was used to assess the contractile function of heart using the 2D-Echo system. 3-lead ECG was recorded during 2D Echo using brass crocodile leads immersed in the perfusate surrounding the heart. Pressures and flow data was acquired and monitored in real time using HMI software, specifically written for SOMAH Device (Comdel Inc, Wahpeton, ND).

### Ex vivo functional studies

Hearts were attached to SOMAH Device and perfused through the aorta at 40-60 mmHg for 5 minutes, with 1-1.5 liters of SOMAH at 21°C (pH 7.5), and then with perfusate, till pH, blood gases and electrolyte equilibrium was established. The perfusate pH, glucose, K^+^, Ca^2+^, HCO^3-^ were adjusted for swine blood levels as mentioned above. Strong cardiac contractions were noted in both groups as the system temperature was gradually raised to 37°C over a 30 minute period. Hemodynamic steady state (with respect to pH, blood gases, and electrolytes) was achieved within 40 minutes. Total duration of the experimental perfusion was approximately 180 minutes. Hearts were perfused through aortic root (no workload) until the system temperature reached 37°C, after which PV perfusion (full workload) proceeded until end of experiment. Coronary blood flow was determined during the initial antegrade perfusion by the amount of perfusate flowing to the heart through aorta per minute, and in the working heart by the amount of perfusate collected from pulmonary artery (both cavas ligated) per minute. Electroconversion (40-50 J) and/or epinephrine (1:50,000-1:100,000) were used if required [14]-[16]. Epicardial 2D Echo was performed using TEE probe for functional assessment at 60 minutes (baseline) and at peak performance, approximately 90 minutes after initiation of perfusate perfusion in the two groups with hearts under full workload; and every 30 minutes thereafter. Peak cardiac performance was defined by the maximum contractile activity observed by 2D Echo. The data at peak performance was used for comparisons between the two groups.

### Enzyme assays and blood chemistry

Quantitative levels of cardiac creatine kinase (CK), aspartate aminotransferase (AST), troponin-I (cTnI), lactate and gases (pO_2_/pCO_2_) were measured intra-operatively and in SOMAH samples taken at 10 minute, 2-hour and at end of 5-hour heart storage using Vetscan VS2 or iStat (Abaxis Ltd, Union City, CA). Inflow (aortic) and outflow (PA) samples were collected for enzyme assays and post perfusion assessment of myocardial O_2_ consumption (MVO_2_) and lactate levels using Vetscan VS2 or i-Stat System, at 5 and 90 minutes for enzyme assays, and at 60 minutes (baseline) and 90 minutes (peak performance) for MVO2 and lactate, after start of perfusate perfusion with Vetscan or iStat. MVO_2_ was calculated as described [20].

### Epicardial echocardiography

A trans-esophageal (TEE) probe was used for 2D echo evaluation of cardiac function intra-operatively and *ex vivo* using the Acuson Cypress system (Acuson, Mountain View, CA) and images were analyzed using Cypress viewer software provided with the system. Hearts were connected to SOMAH Device and suspended in a chamber containing 2 L of perfusate that covered 2/3^rd^ surface of the heart. ECG was recorded during entire course of the experiment and 2D Echo acquisition was begun approximately 45-60 minute after perfusion, when good cardiac contractions were observed, and repeated at 30-minute intervals. Probe was placed in direct contact with heart and angle of probe and direction of pulse were adjusted as to obtain short-axis and long-axis views for calculations of cardiac functional parameters, and ventricular wall and septal thickness.

### Statistical analyses

Equal number of animals (n = 5), were assigned to 4°C and 21°C cardioplegia groups for comparative analysis for biochemical, hemodynamic and functional measurements from each group. Statistical comparison for significant differences between the two groups was performed using SigmaPlot software. Paired t-test was used for all comparisons. P-value of <0.05 was considered significant. All values are expressed as mean ± SEM. Flow diagram of the experimental design is shown in Figure 2.

The authors had full access to the data and take full responsibility for its integrity. All authors have read and agreed to the manuscript as written. The funding agencies did not play any role in influencing data collection, extraction and interpretation.

## Results

### Intraoperative cardioplegia

Cardiac arrest was dependent on the temperature of cardioplegia and occurred within 10-15 seconds in 4°C group and 20-25 seconds in the 21°C group, likely because the hypothermic (4°C) component of cardiac arrest was deliberately eliminated in the latter.

### Gross morphology, heart weights and release of enzymes during storage

Irrespective of temperature of cardioplegia, all hearts presented normal gross morphology without any discoloration. Hearts were pliable with no signs of hardening or stiffness. Weights of the hearts during 5-hour storage in the two groups were not altered between pre and post storage, demonstrating lack of storage-induced gross edema (not shown). A time dependent release of cardiac enzymes was minimally apparent in both groups that were not significantly different (not shown).

### Cardiac tissue HEP levels after arrest and during storage

**Figure 3:**
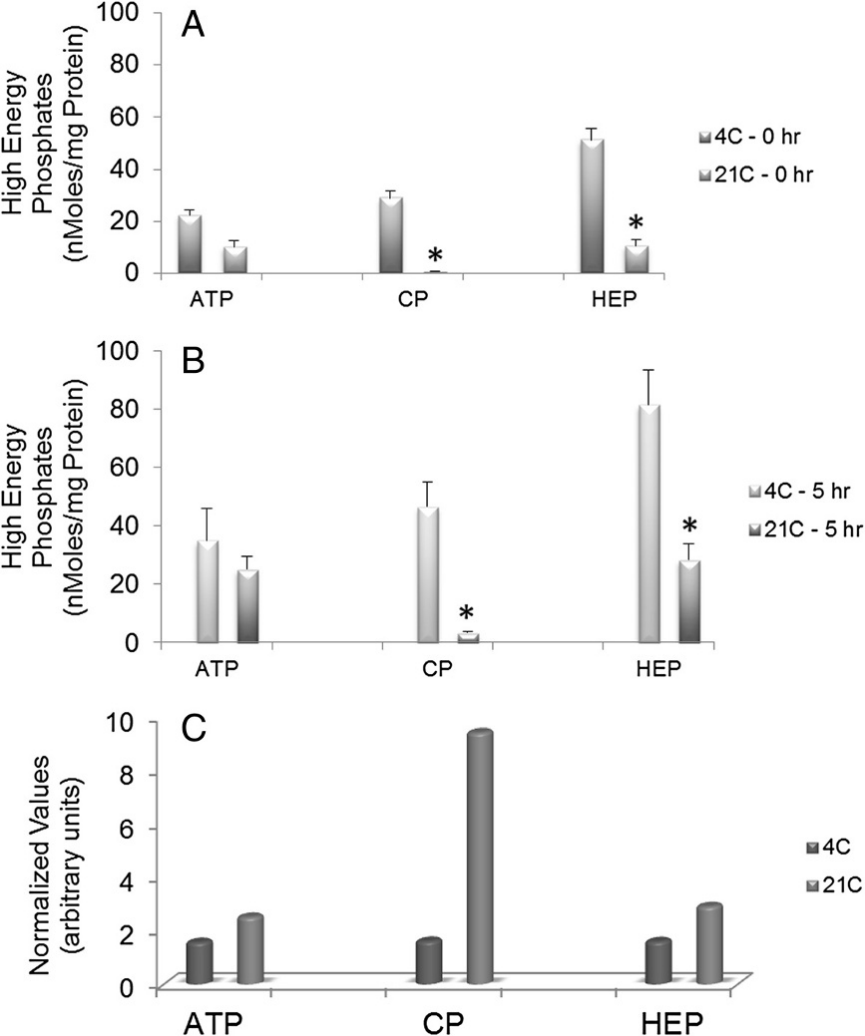
***High energy phosphate levels in hearts arrested and stored in SOMAH.*** Cardiac tissue biopsies from left ventricle were obtained for determination of HEP including ATP and CP levels in 4 and 21°C SOMAH cardioplegia group hearts pre and post 5-hour storage. There was a temperature of cardioplegia arrest dependent increase in HEP concentrations in the hearts. **A**: Control; **B**: 5 hour storage; **C**: Normalized values (5 hours with respect to 0 hour). Each bar represents mean ± SEM of n = 5 for each group. * Significantly different from 4°C cardioplegia group hearts.

As shown in Figure 3, concentration of ATP, CP and total high-energy phosphates (HEP), within 15 minutes (control) of cardiac arrest were significantly greater in 4°C as compared to 21°C cardioplegia hearts (P < 0.001) possibly due to the greater consumption of energy in 21°C group hearts as they required approximately 10 seconds longer for total arrest. Both groups actively synthesized CP and ATP during storage, Figure 3B. While the total concentrations of HEP at the end of storage were significantly greater in 4°C hearts (P < 0.01), normalization of values at 5 hour with respect to those at 0 hour demonstrated greater availability of HEP in 21°C hearts than in 4°C hearts at the end of storage, Figure 3C.

### Ex vivo cardiac functional studies

#### Coronary flow upon reperfusion

Coronary flow through the aorta, upon initial antegrade perfusion at similar perfusion pressures, was significantly greater in 21°C hearts (P < 0.05) than the 4°C group (Table 1). The hearts in both groups demonstrated slow four chamber contractions immediately upon initiation of perfusion. Coronary flow decreased initially when system temperature was raised to 30°C, as the hearts started contracting vigorously. Both pressure and flow increased as system temperature was raised to 37°C. Coronary flow was highest in both groups at 37°C (Table 1).

**Table 1:** **Coronary flow (ml/min) in differential temperature cardioplegia hearts with the rise in system temperature of SOMAH Device**

Heart groups	P1 - 21°C	F1 - 21°C	P2 - 30°C	F2 - 30°C	P3 - 37°C	F3 - 37°C
**4°C**	40 ± 1	312 ± 90	44 ± 2	297 ± 28	53 ± 4	621 ± 88
**21°C**	40 ± 1	464 ± 65*	40 ± 2	348 ± 64	50 ± 3	619 ± 34

P1, P2, P3 - Aortic Root Pressures at respective temperatures.

F1, F2, F3 - Coronary Flows at respective temperatures.

*Significant from 4°C.

#### Release of enzymes upon reperfusion

**Figure 4:**
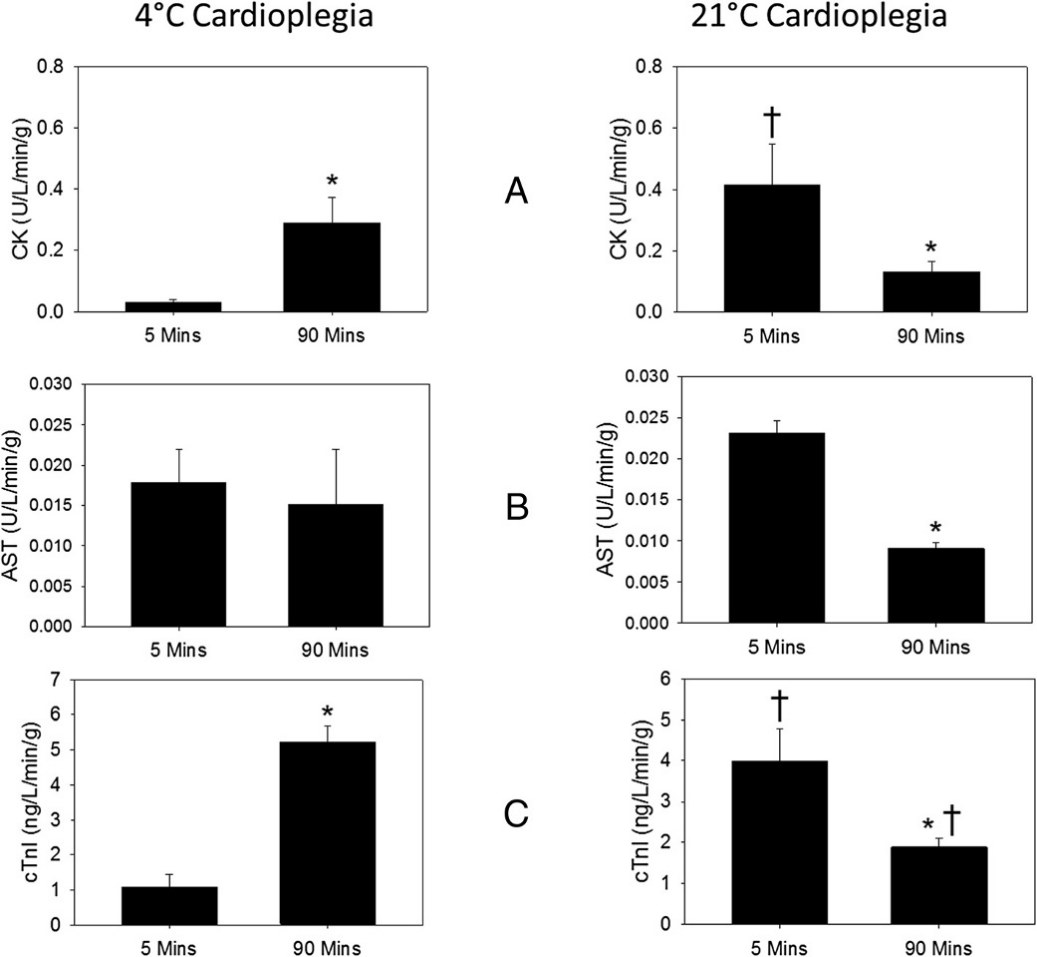
***Release of creatine kinase and cardiac troponin-I upon reperfusion.*** CK **(A)**, AST **(B)** and cTnI **(C)** levels were determined in perfusate, 5 min and 90 min (peak performance) after start of reperfusion of 4 and 21°C cardioplegia hearts; n = 5 for each SOMAH group. *Significant change from 5 minutes (p <0.05); * Significantly different from 4°C cardioplegia group hearts at similar time point.

Rate of CK, AST and cTnI release increased in 4°C hearts during the perfusion period (Figure 4). Both CK and cTnI release increased significantly with the time of perfusion, but the AST did not. In contrast, there was a temporal decrease in release of these three enzymes in the 21°C hearts during the same period (Figure 4). However, release of CK and cTnI, but not the AST, was significantly greater by the 21°C hearts upon initiation of perfusion than by the 4°C hearts.

#### Metabolism in reperfused hearts

**Figure 5:**
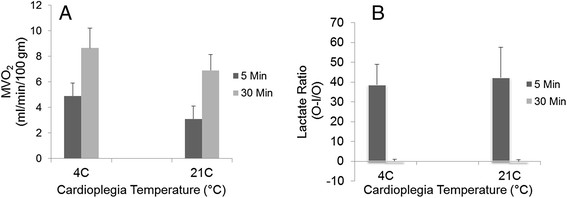
***Cardiac metabolism in working hearts*** **.** Myocardial O_2_ Consumption **(A)** and Lactate Ratio **(B)** upon perfusion of 4 and 21°C cardioplegia hearts. MVO_2_ and Lactate ratios were determined from the differences in the respective parameters in the outflow and inflow perfusate samples. Baseline = 60 min after reperfusion, at hemodynamic steady state; 90 min = at peak performance. Each bar represents mean ± SEM of n = 5 for each group.

Consistent with our previous observations [14]-[16], there was a rapid switch in metabolism from anaerobic to aerobic upon reperfusion in both 4°C and 21°C cardioplegia groups, demonstrated by an increase in oxygen consumption and reversal of lactate ratios at peak performance, 90 minutes into reperfusion (Figures 5A and B). Oxygen extraction, lactate production and utilization, reached a steady state in both groups at peak performance that were not significantly different. However, upon reperfusion, 21°C group hearts demonstrated robust synthesis of HEP in the working hearts. In contrast, production was attenuated in 4°C hearts and the HEP continued to decline during course of the experiment. Ratios (post perfusion/pre perfusion) of ATP, CP and total HEP were 1.10, 1.97 and 1.17, respectively in 21°C hearts which were significantly greater (p < 0.01) than ratios of 0.47, 0.32 and 0.38 observed in the 4°C hearts at end of experiment, when post perfusion biopsies were taken for HEP assays.

#### Functional revival upon reperfusion

Immediate spontaneous activity of both the atria and ventricles was apparent upon commencement of reperfusion in both groups. With increased temperature, force of ventricular contraction peaked at about 37°C after a single cardioversion, which also established the normal electrical activity and electromechanical coupling in sinus rhythm in both groups. While 4 of the 5 hearts in 21°C cardioplegia group reverted to sinus rhythm with a single cardioversion and none requiring further inotropic support, 2 of the 5 hearts in 4°C cardioplegia group required additionally a single dose of epinephrine to maintain optimal function. Interestingly, hearts in both groups remained pliable throughout the experiment and did not show any edema at peak performance as indicated by unaltered thickness of LV and the septum, Table 2. The comparative data between 4 and 21°C cardioplegia groups for cardiac functional parameters acquired by 2D Echo were similar to those observed *in vivo* (Table 2). Although hearts that received SOMAH cardioplegia at 21°C appeared to have a better recovery, there were no significant differences in the functional parameters of the two groups.

**Table 2:** **Cardiac functional parameters during surgery and at peak performance upon extracorporeal reperfusion in 4°C and 21°C SOMAH cardioplegia group hearts**

Parameters	In vivo	4°C hearts	21°C hearts
**LV ant wall thickness (cm)**	1.48 ± 0.07	1.55 ± 0.06	1.51 ± 0.04
**Septal thickness (cm)**	1.43 ± 0.12	1.61 ± 0.04	1.49 ± 0.07
**Heart rate**	98 ± 12	100 ± 10	110 ± 10
**LV systolic pressure (mm Hg)**	116 ± 8	100 ± 20	110 ± 10
**LV diastolic pressure (mm Hg)**	68 ± 7	40 ± 10	55 ± 10
**Left atrial pressure (mm Hg)**	<10	0-5	0-5
**Cardiac output**	3300 ± 400	2500 ± 300	2640 ± 250
**Fractional area change (%)**	>40	42 ± 13	51 ± 4
**Ejection fraction (%)**	65 ± 5	58 ± 4	66 ± 5
**Stroke volume (ml)**	29 ± 4	25 ± 2	24 ± 3

## Discussion

We have designed and evaluated SOMAH, a synergistic extracellular organ storage solution that includes various energy substrates, metabolic modulators, free radical scavengers and anti-oxidants, ammonia chelators and nitric oxide synthase substrates, intra and extracellular H^+^ chelators and physiological concentration of calcium, components that help prevent edema, ionic imbalance and energy depletion [12]. SOMAH provides a favorable environment and cellular support during ex-vivo storage of hearts, lungs and abdominal organs, and helps attenuate reperfusion injury upon reanimation [13]-[16],[21]. Promising results with organ preservation encouraged us to undertake this study to evaluate SOMAH as a cardioplegia solution.

Recent results from our laboratory show that extracorporeal preservation of hearts for transplant at 21°C (ambient temperatures) in SOMAH is ideal for the highest promotion of synthesis and accumulation of HEP in heart tissue and preservation of ideal function [13]-[16]. Based on these results and on the reported advantages of crystalloid solution [4],[22] and tepid temperatures for cardioplegia [15], the present study was undertaken to evaluate whether using crystalloid SOMAH at 21°C instead of standard temperature of 4°C for cardioplegia could further improve the quality of hearts preserved at ambient temperature in SOMAH, and their eventual reanimation *in vitro* into optimal function.

Myocardial edema has been reported in hearts arrested at perfusion pressures of cardioplegia as low as 50 mmHg [23]. However, in present and past studies we have consistently used a crystalloid SOMAH cardioplegia infusion pressure of 100 mmHg irrespective of cardioplegia temperature, but edema was not seen in any of the hearts [14]-[16]. SOMAH cardioplegia provides all the advantages of blood cardioplegia, in terms of protection from cardiac edema and provision of substrates for energy metabolism, and also provides clear surgical field. Additionally, provision of physiological concentrations of calcium in SOMAH solution, also prevents the likelihood of myocardial damage due to `calcium paradox' [6],[24]. Furthermore, the disadvantages of blood such as the presence of leukocytes and platelets, culpable in reperfusion injury [25], are also averted. In contrast, the other crystalloid solutions (Celsior and UWS), when used for cardioplegia in our recent studies did not prevent loss of high-energy phosphates in the stored hearts, edema upon reperfusion and potential high K^+^ mediated calcium overload and stiffness that resulted in non-functioning hearts [14]-[16].

In this study, HEP were conserved in 4°C hearts because of rapid arrest, as a result total concentration of HEP was also greater in these hearts at the end of storage. In contrast, despite K^+^ concentration (20 mM) being equal in the two groups, the 21°C hearts took longer for total arrest because of absence of the hypothermic component, resulting in depletion of HEP. Both groups synthesized HEP during storage in SOMAH, however, the functional availability of HEP was greater in 21°C hearts than the 4°C hearts at the end of 5 hour (Figure 3). Similarly, upon reperfusion, 21°C hearts continued to synthesize HEP to meet the demands of the working heart, unlike the 4°C hearts. At peak performance the available HEP in 21°C hearts was significantly greater than 4°C hearts, and continued to be so during course of the experiments. In contrast, 4°C hearts were unable to synthesize HEP to keep with the energy demands, thus HEP continued to decrease in the working hearts. These results are in agreement with our previous observations that unlike in hearts preserved in SOMAH at ambient temperatures, exposure to severe hypothermia leads to attenuation of HEP synthesis upon reperfusion in these hearts [13].

Antegrade perfusion was significantly lower in the 4°C heart than in 21°C hearts, and remained diminished, even at higher perfusion pressures until the system temperature stabilized at 37°C (Table 1). It is plausible that sudden shock of encountering 4°C cardioplegia by the normothermic beating heart leads to profound vasoconstriction that does not resolve during storage and only does so upon initiation of reperfusion and raising of temperature to 37°C, and potentially because of active release of vasodilators nitric oxide and prostacyclins [12]. Increased vasodilation, greater coronary vascular patency and a favorable metabolic status provides for rapid nourishment and H^+^ washout, resulting in robust synthesis of HEP and swift recovery of function in the 21°C hearts. These hearts reverted to sinus rhythm with a single cardioversion and rapidly attained cardiac and hemodynamic parameters approaching *in vivo* range (Table 2), not requiring any inotropic support. On the other hand, 4°C hearts demonstrated strong contraction only when warmed to 37°C, some of the hearts requiring additional electroversion and/or inotropic intervention, albeit ten times less than that reported in human hearts *in vitro*[26] to maintain cardiac output.

Release of cardiac enzymes was observed in both the groups upon reperfusion. An important mechanism of release of enzymes from the cardiomyocyte is by cytosol leakage during intracellular vesicular trafficking and incorporation (such as vesicles harboring glucose transporters; GLUT) into cell membrane, by a HEP dependent process, in response to external stimuli like insulin and increased metabolic demands in working hearts [27]. Therefore, release of cellular enzymes can occur even in absence of actual damage to the cardiomyocytes (enzyme paradox). Initial burst of enzyme release in 21°C cardioplegia group upon initiation of reperfusion likely resulted from greater availability of HEP at the end of storage for vesicular transport as the metabolic demands were ramped up with increase in system temperature and cardiac contractility (Figure 3). However, upon reaching metabolic steady state any further release of enzyme was temporally attenuated. In contrast, in the 4°C hearts, the rate of release of enzymes increased with time as more HEP became available for these functions. Even though the present data does not differentiate between progressions of enzyme release and needs further investigation, the fact, that the cardiac functional parameters in both groups were similar and approach the physiological values observed *in vivo* (Table 2) indicate that the releases of enzymes by the SOMAH hearts is a marker of metabolism rather than tissue injury and hence these hearts would perform well upon transplant.

Certain limitations were inherent to this study. While the present findings indicate a certain advantage of using SOMAH at 21°C for clinical cardioplegia either intraoperatively or during heart procurement for transplantation, in the present preliminary study we have performed only a modest number of experiments in each group and used 2D echocardiography for the functional evaluation of reperfused hearts. While this provided convincing evidence in favor of 21°C cardioplegia, further studies using Pressure-Volume loops acquisition and analysis for precise determination and interpretation of the significant bio-physiological effects of SOMAH on extracorporeal cardiac reperfusion under varying conditions, are required.

## Conclusion

Efficient HEP metabolism, decreased rate of enzyme release, and diminished requirement for stimulatory interventions suggest that use of SOMAH for cardioplegia and storage at 21°C optimally protects the heart function and thus may positively influence post operative outcomes upon transplant. Extrapolating ex vivo 5 hour preservation as analogous to cross clamp time, use of SOMAH cardioplegia at ambient temperatures during open heart surgery, where the cross clamp times generally do not exceed 3 hours (average 60-90 minutes); where the hearts are periodically flushed with 500 ml of fresh cardioplegia every 20 to 30 minutes during cross clamp, may significantly improve post surgical outcomes. However, further studies are needed to translate this inferential hypothesis into a clinical reality.

## Authors' contributions

SKL and HST designed the experiments and analyzed the data. SKL acquired the 2D Echo data intraoperatively and ex vivo, conducted the experiments and wrote the manuscript. HST performed the heart procurement surgeries, helped in overall conduct of experiments and preparation of the manuscript. PRT assisted in infusion of cardioplegia, set up of ex vivo perfusion apparatus and in conduct of experiments. All authors read and approved the final manuscript.

## Additional file

## Electronic supplementary material

Additional file 1: Somah Device - Representative of a Working Heart. (ZIP 9 MB)

Below are the links to the authors’ original submitted files for images.

Authors’ original file for figure 1

Authors’ original file for figure 2

Authors’ original file for figure 3

Authors’ original file for figure 4

Authors’ original file for figure 5

## Abbreviations

2D Echo: 2 Dimensional echocardiography
AST: Aspartate aminotransferase
ATP: Adenosine tri-phosphate
CK: Creatine kinase
CP: Creatine phosphate
cTnI: Troponin-I
ECG: Electrocardiograph
HEP: High energy phosphate
MVO_2_: Myocardial O_2_ consumption
PA: Pulmonary artery
PV: Pulmonary vein
TEE: Trans-esophageal echocardiography
UWS: University of Wisconsin Solution
