# Supplementary figures and images for: Cardioplegia at subnormothermia facilitates rapid functional resuscitation of hearts preserved in SOMAH for transplants
Source: J Cardiothorac Surg. 2014 Sep 20;9:155. doi: 10.1186/s13019-014-0155-z (PMC4182865; doi:10.1186/s13019-014-0155-z)

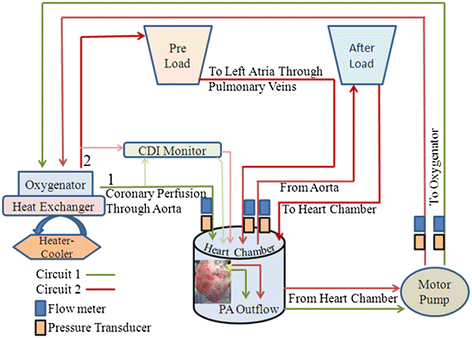

Supplement: Supplementary file 2 — Authors’ original file for figure 1 [file 13019_2014_155_MOESM2_ESM.gif]

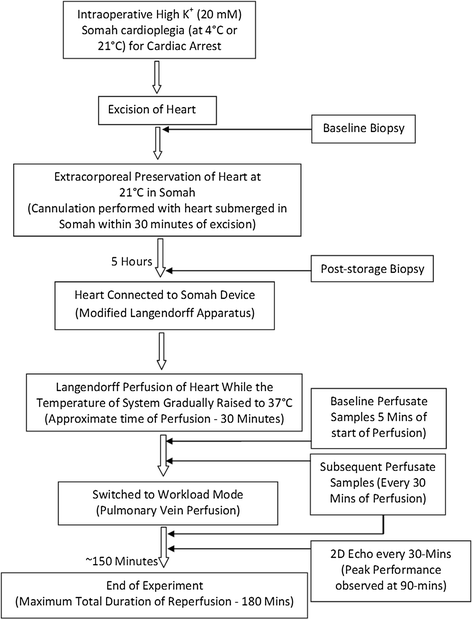

Supplement: Supplementary file 3 — Authors’ original file for figure 2 [file 13019_2014_155_MOESM3_ESM.gif]

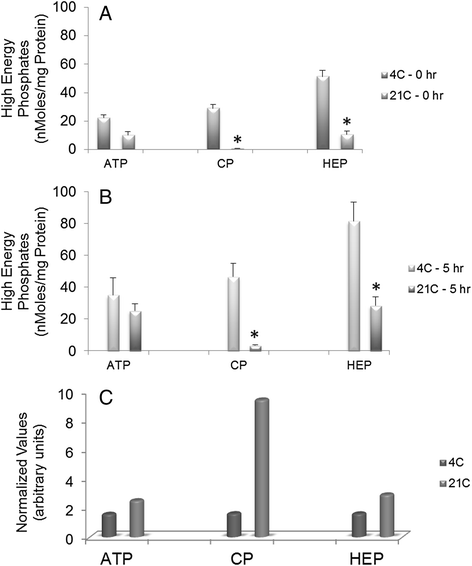

Supplement: Supplementary file 4 — Authors’ original file for figure 3 [file 13019_2014_155_MOESM4_ESM.gif]

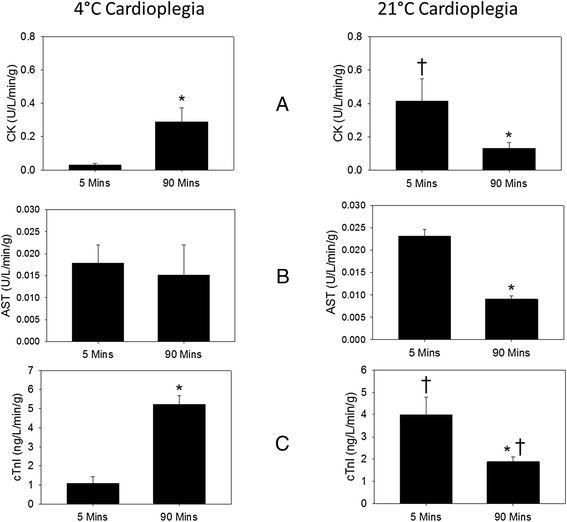

Supplement: Supplementary file 5 — Authors’ original file for figure 4 [file 13019_2014_155_MOESM5_ESM.gif]

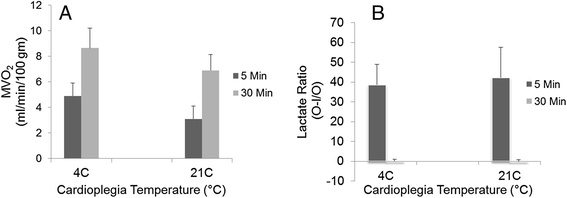

Supplement: Supplementary file 6 — Authors’ original file for figure 5 [file 13019_2014_155_MOESM6_ESM.gif]
